# O-linked β-N-acetylglucosamine modification in diabetic foot ulcer pathogenesis

**DOI:** 10.1093/burnst/tkaf044

**Published:** 2025-07-14

**Authors:** Bingxue Qi, Siyang Chai, Yang Chen, Guannan Zhou, Peihong Li, Xueqing Li, Xiaodan Lu, Li-Hao Huang

**Affiliations:** Precision Molecular Medicine Center, Jilin Province People's Hospital, 1183 Gongnong Road, Chaoyang District, Changchun 130021, China; Clinical Medicine College, Changchun University of Chinese Medicine, 1035 Boshuo Road, Jingyue District, Changchun 130117, China; Clinical Medicine College, Changchun University of Chinese Medicine, 1035 Boshuo Road, Jingyue District, Changchun 130117, China; Department of Gynecology, The Obstetrics and Gynecology Hospital of Fudan University, 419 Fang-Xie Road, Huangpu District, Shanghai 200011, China; Clinical Medicine College, Changchun University of Chinese Medicine, 1035 Boshuo Road, Jingyue District, Changchun 130117, China; Clinical Medicine College, Changchun University of Chinese Medicine, 1035 Boshuo Road, Jingyue District, Changchun 130117, China; Precision Molecular Medicine Center, Jilin Province People's Hospital, 1183 Gongnong Road, Chaoyang District, Changchun 130021, China; Shanghai Key Laboratory of Metabolic Remodeling and Health, Institute of Metabolism and Integrative Biology, Liver Cancer Institute, Zhongshan Hospital, Fudan University, 2005 Songhu,Yangpu District, Shanghai 200438, China

**Keywords:** O-linked β-D-N-acetylglucosamine modification, Diabetic foot ulcers, Pathogenesis, peripheral neuropathy, Peripheral arterial disease, Wound healing, Metabolic dysregulation, therapeutic target

## Abstract

O-linked β-D-N-acetylglucosamine (O-GlcNAc) modification represents a common form of posttranslational glycosylation orchestrated by two pivotal enzymes, namely, O-GlcNAc transferase and O-GlcNAcase. In recent years, emerging research has revealed a significant association between O-GlcNAc modification and the pathogenesis of diabetic foot ulcers (DFUs). Elevated O-GlcNAc levels under high-glucose conditions contribute to the pathogenesis of DFUs by modifying specific proteins, which are implicated in peripheral neuropathy, peripheral vascular disease, and impaired chronic wound healing. This process includes prolonged inflammation, compromised granulation tissue formation, disordered re-epithelialization, and blocked tissue remodelling. This review focuses on the pathogenesis of DFUs and on the correlation between protein O-GlcNAc modification and DFUs, offering potential new insights for the diagnosis and treatment of this condition.

## Graphical Abstract

**Graphical Abstract:**
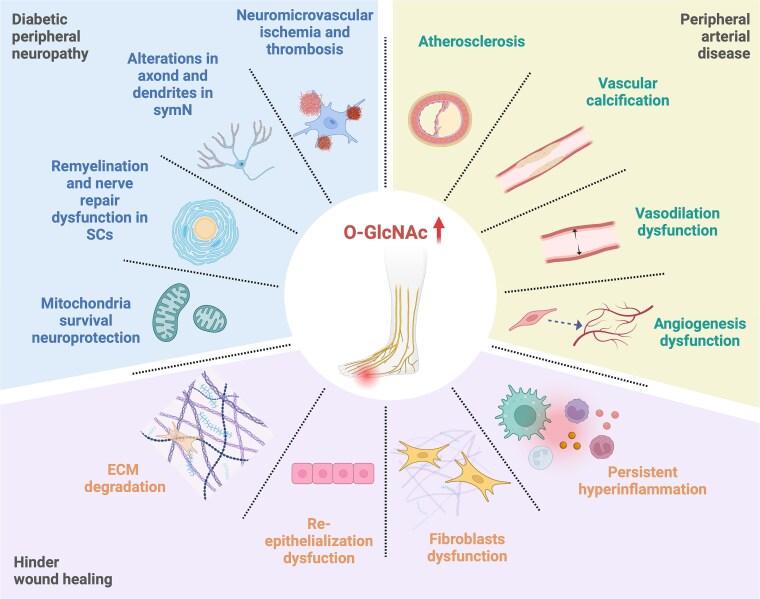

Highlights

- Elevated O-linked β-D-N-acetylglucosamine (O-GlcNAc) modification under high-glucose conditions contributes to diabetic foot ulcers (DFUs) pathogenesis by altering specific proteins involved in peripheral neuropathy, peripheral vascular disease, and chronic impaired wound healing.
- O-GlcNAc modification impacts distinct phases of the DFU healing process, including inflammation, granulation, and re-epithelialization.
- O-GlcNAc modification presents potential molecular targets for developing novel treatments for DFUs.

## Background

Diabetes mellitus (DM) is a metabolic disorder characterized by inadequate insulin secretion or insulin resistance. The International Diabetes Federation anticipates a significant increase in the global prevalence of diabetes, forecasting an increase to 10.9% by 2045, which will affect ~700 million individuals [1]. Among the complications associated with diabetes, diabetic foot ulcers (DFUs) are particularly severe, often resulting in chronic, nonhealing wounds and representing a leading cause of lower limb amputations in diabetic patients [2]. DFUs occur in 19%–30% of diabetic patients worldwide, and if untreated, they can escalate to soft tissue infections, gangrene, and limb loss [3]. The financial burden of treating DFUs is substantial [4]. Therefore, increasing awareness of DFUs, along with early prevention, identification, and intervention, is crucial for delaying disease progression, reducing the economic burden on patients, and lowering amputation and mortality rates.

The management of DFUs relies on a comprehensive treatment strategy that includes blood glucose control, nutritional support, infection management, diabetic peripheral neuropathy (DPN) treatment, peripheral artery disease (PAD) treatment, vascular interventions, foot pressure reduction, and hyperbaric oxygen therapy. Wound management, aimed at promoting healing and reducing inflammation, is central to DFU care and involves the use of advanced dressings, negative pressure wound therapy, skin grafts, and various biological therapies, such as stem cell treatment and immunomodulatory therapies [5–8]. Despite these advancements, the traditional methods are insufficient for complete treatment of DFUs, owing to their complex pathogenesis. The pathogenesis of DFUs is complex and involves multiple factors, with diabetic PAD and DPN being major contributors [9]. Normal wound healing is a sophisticated physiological process that typically encompasses four key stages—haemostasis, inflammation, proliferation, and tissue remodelling. Failure of any of the above stages may lead to difficult or even impossible skin wound healing [10]. Hyperglycaemia increases the risk of infection, as various mechanisms sustain a state of heightened inflammation in the wound [11, 12]. Prolonged inflammation, compromised granulation tissue formation, impaired re-epithelialization, and blocked remodelling collectively contribute to the delayed healing of DFU wounds [13]. Single nucleotide polymorphisms and phosphorylation modifications also play important roles in the pathogenesis of DFUs [14, 15]. DFUs are notoriously challenging to manage clinically because of the multifaceted mechanisms underlying their development and persistence. These factors exacerbate the occurrence and persistence of ulcers by influencing cellular function, inflammatory responses, and tissue repair capacity, thereby complicating the healing process and increasing the risk of chronic complications.

N-acetylglucosamine (GlcNAc) modifications can be divided into two main types—O-linked β-N-acetylglucosamine (O-GlcNAc) modifications and N-linked β-N-acetylglucosamine (N-GlcNAc) modifications. Both types of GlcNAc modifications play significant roles in regulating protein function, cellular signal transduction, and the occurrence of diseases, but their mechanisms of action and biological significance differ. O-GlcNAc modification is more closely associated with intracellular metabolism and signal transduction, whereas N-GlcNAc modification is related to protein folding, stability, and functions at the cell surface. In this review, we focused on the role and mechanisms of O-GlcNAc modification in DFUs. In 1984, Hart and colleagues first described a modification of proteins involving the attachment of a single GlcNAc molecule to serine (Ser) and threonine (Thr) residues via an O-linkage, and they named this modification O-GlcNAc modification [16]. O-GlcNAc modification is a prevalent and dynamic posttranslational modification (PTM) that occurs in the nucleus, cytoplasm, and mitochondria. GlcNAc modification plays a vital role in a multitude of biological processes, such as gene transcription, signal transduction, epigenetic modifications, nutrient sensing, the stress response, metabolic homeostasis, and the immune response [17]. O-GlcNAc modification is increasingly recognized as an important cellular metabolic and signalling regulatory mechanism closely related to the development of diabetes and its complications. In recent years, research has shown that O-GlcNAc modification also plays a significant role in diabetic complications, such as diabetic cognitive dysfunction, diabetic retinopathy, and diabetic nephropathy [18–20]. Notably, O-GlcNAc modification is a key regulator of the impaired wound-healing process observed in diabetic wounds, contributing to the formation and persistence of chronic wounds in DFUs. An increase in O-GlcNAc modification may contribute to the development of DPN by causing microvascular ischaemia and impairing nerve cell function [21–23]. Furthermore, elevated levels of O-GlcNAc modification promote atherosclerosis and impair vascular function [24, 25]. Additionally, studies have shown that O-GlcNAc modification affects the proliferation and migration of cells involved in wound healing, such as keratinocytes and fibroblasts. For example, hyper-O-GlcNAc modification of c-Myc in keratinocytes has been shown to impair their function, leading to delayed wound closure [26]. Similarly, in endothelial cells, O-GlcNAc modification alters the expression of genes involved in cell migration and proliferation, affecting the overall wound-healing process [27]. Elevated O-GlcNAc levels are associated with increased activation of transcription factors and signalling pathways that promote inflammation and oxidative damage, further complicating the healing process [28]. Although numerous studies have explored the role of O-GlcNAc modification in DFUs, there is still a lack of understanding of its specific mechanisms during wound healing. This review summarizes the current understanding of the roles and regulatory mechanisms of O-GlcNAc modification in DFUs, highlighting the potential application of O-GlcNAc modification-targeted therapy in DFUs.

## Review

### Molecular regulation of O-GlcNAc modification

**Figure 1:**
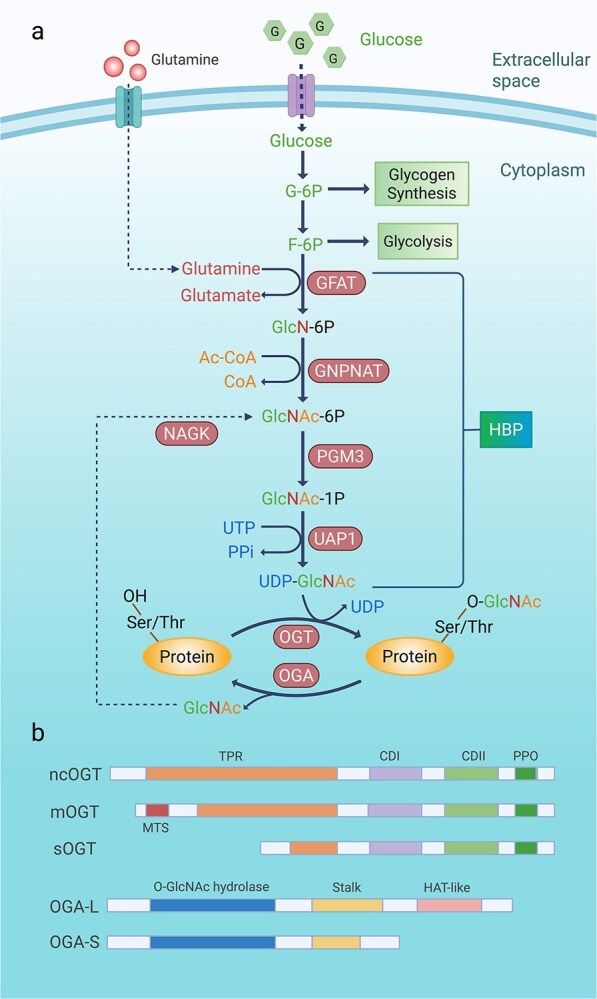
The molecular regulation of the HBP and O-GlcNAc modification. (**a**) the HBP is a minor branch of glycolysis, where glucose is converted to F-6P through the initial steps shared with glycolysis. Under normal conditions, 2%–5% of glucose is shunted into the HBP. Glucose is rapidly phosphorylated to G-6P by hexokinase and then isomerized to F-6P. The rate-limiting step of HBP involves the conversion of glutamine and F-6P into GlcN-6P by GFAT. Subsequent reactions include the formation of GlcNAc-6P and GlcNAc-1P, culminating in the production of UDP-GlcNAc. UDP-GlcNAc is used by OGT to attach GlcNAc to Ser or Thr residues on proteins, forming O-GlcNAc glycosylation. OGA reverses this modification. GlcNAc can re-enter the HBP via a recycling pathway mediated by NAGK. (**b**) O-GlcNAc modification is regulated by OGT and OGA, which add and remove GlcNAc, respectively. OGT has three isoforms: ncOGT, mOGT, and sOGT, differing in their TPR domain length but sharing common catalytic and phospholipid-binding domains. mOGT has a unique MTS. OGA has two isoforms, OGA-L and OGA-S, sharing the same N-terminal hydrolase domain and OGT binding region, but OGA-S lacks the C-terminal HAT-like domain and part of the stalk domain found in OGA-L. *HBP* hexosamine biosynthetic pathway, *G-6P* glucose-6-phosphate, *F-6P* fructose-6- phosphate, *GlcN-6P* glutamate and glucosamine-6-phosphate, *GFAT* glutamine fructose-6-phosphate aminotransferase, *GNPNAT* glucosamine-phosphate N-acetyltransferase, *PGM3* phosphoglucomutase 3, *GlcNAc-6P* p N-acetylglucosamine-6-phosphate, *GlcNAc-1P* N-acetylglucosamine-1-phosphate, *UAP1* uDP-N-acetylglucosamine pyrophosphorylase 1, *PPi* pyrophosphate, *OGT* o-GlcNAc transferase, *OGA* o-GlcNAcase, *Ser* serine, *Thr* threonine, *NAGK* N-acetylglucosamine kinase, *ncOGT* nucleocytoplasmic OGT, *mOGT* mitochondrial OGT; *sOGT* short OGT, *TPR* Tetratricopeptide repeat, *CDI and CDII* Carboxy-terminal (C-terminal) catalytic, *PPO* phosphoinositide-binding domains, *MTS* mitochondrial targeting sequence, *OGA-L* long OGA isoforms, *OGA-S* short OGA isoforms, *HAT-like* histone acetyltransferase-like

The hexosamine biosynthesis pathway (HBP) is a critical metabolic route that converts glucose and other metabolites into UDP-GlcNAc, essential substrate that is pivotal for O-GlcNAc modification (Figure 1a). Under normal physiological conditions, ~2% to 5% of glucose enters the HBP. However, under hyperglycaemic conditions, this percentage significantly increases as more glucose is shunted towards the HBP [29]. Once inside the cell, glucose undergoes isomerization to fructose-6-phosphate (F-6P) through enzymatic catalysis. The first and rate-limiting step of the HBP is the conversion of glutamine and F-6P into glutamate and glucosamine-6-phosphate (GlcN-6P) by the glutamine fructose-6-phosphate aminotransferase (GFAT) rate-limiting enzyme. Glucosamine-phosphate N-acetyltransferase (GNPNAT) then converts GlcN-6P and acetyl-coenzyme A into N-acetylglucosamine-6-phosphate (GlcNAc-6P) and CoA. GlcNAc-6P is then converted to N-acetylglucosamine-1-phosphate (GlcNAc-1P) by phosphoglucomutase 3 (PGM3). The final step involves UDP-N-acetylglucosamine pyrophosphorylase 1 (UAP1), catalyzing the reaction of GlcNAc-1P with UTP to generate UDP-GlcNAc and pyrophosphate (PPi) [30]. UDP-GlcNAc serves as a substrate for OGT, which attaches GlcNAc monosaccharides to the Ser or Thr residues of the target protein to form O-GlcNAc modifications. Conversely, OGA removes GlcNAc monosaccharides from proteins, enabling the dynamic regulation of protein O-GlcNAc modification [31]. GlcNAc can re-enter the HBP via the recycling pathway mediated by N-acetylglucosamine kinase (NAGK).

OGT is the only glycosyltransferase found in the nucleus and cytoplasm of mammals and is expressed mainly in the nucleus (Figure 1b). OGT comprises an N-terminal domain containing 13 tetratricopeptide repeat (TPR) sequences and a C-terminal catalytic domain. The TPR domain of OGT regulates its interactions with target and regulatory proteins, and it plays a direct role in substrate selection [32]. The C-terminus of OGT is the catalytic domain, which includes a UDP-GlcNAc-binding site and an enzyme catalytic site [33]. The OGT gene encodes three different isoforms as follows: nucleocytoplasmic OGT (ncOGT), which consists of 13.5 TPRs, has a molecular weight of 116 kD, and is present in both the cytoplasm and nucleus; mitochondrial OGT (mOGT), which consists of 9.5 TPRs, has a molecular weight of 103 kD, and it present only in the mitochondria; and short OGT (sOGT), which consists of 2.5 TPRs, has a molecular weight of 70 kD, and is present in the nucleus [34]. These isomorphs differ in the length of the TPR domain but share common carboxy-terminal (C-terminal) catalytic (CDI and II) and phosphoinositide-binding (PPO) domains. mOGT contains a unique N-terminal mitochondrial targeting sequence (MTS) [31]. The majority of research on OGT substrate selectivity has been conducted with ncOGT, which is a major player in intracellular O-GlcNAc modification [35].

OGA encodes two distinct splice variants referred to as long (OGA-L) and short (OGA-S) isoforms (Figure 1b). OGA-L consists of three connected domains—an N-terminal O-GlcNAc hydrolase domain, an intermediate stalk domain, and a C-terminal acetyltransferase domain [36]. The N-terminal domain is the catalytic domain, which has OGA activity and is responsible for removing O-GlcNAc modifications from its target proteins. The C-terminal domain is known as the HAT-like or histone acetyltransferase-like domain, and it is involved in histone regulation [37]. OGA-L, with a molecular weight of 102 kDa, is distributed in the cytoplasm and nucleus, whereas OGA-S, with a molecular weight of 76 kDa, is distributed in the sarcoplasmic reticulum and lipid droplets. Unlike OGA-L, OGA-S lacks the C-terminal acetyltransferase domain and is part of the stalk domain. OGA-S overexpression has been shown to increase mitochondrial reactive oxygen species (ROS) levels [38]. Like OGT, OGA is highly conserved and ubiquitously expressed across tissues, with higher levels observed in the pancreas, brain, and skeletal muscle [39]. OGT and OGA interact and coordinate to enable organisms to respond to external or internal stimuli by rapidly controlling protein O-GlcNAc glycosylation levels, but the involved complex mechanisms remain poorly defined.

Protein phosphorylation is the most abundant PTM in proteins. More than 500 kinases phosphorylate their target proteins with obvious sequence specificity [40]. It is widely believed that dynamic mutual regulation between O-GlcNAc glycosylation and phosphorylation jointly mediates cellular functions; the modification sites are all Ser and Thr residues, and different enzymes precisely regulate them [41]. Unlike phosphorylation, which is regulated by various phosphates and protein kinases, O-GlcNAc glycosylation is regulated by only two enzymes, namely, OGT and OGA, which are responsible for GlcNAc addition and removal, respectively, in mammalian cells [42]. Notably, O-GlcNAc glycosylation does not have a strict amino acid consensus sequence, which may allow it to interact with phosphorylation more complexly. In the context of DFUs, the interplay between O-GlcNAc glycosylation and phosphorylation is particularly intriguing. Recent studies have suggested that these modifications have competitive or synergistic effects on protein function, which may significantly impact the pathology of DFUs. For example, O-GlcNAc modification interferes with phosphorylation by blocking key protein phosphorylation sites, thereby altering protein activity and downstream signalling pathways [43]. Conversely, phosphorylation also regulates O-GlcNAc glycosylation by modulating the activity of OGT or OGA [44]. The wound environment in DFUs is marked by extensive metabolic remodelling, which may further complicate the interplay between these modifications. Elevated metabolites, such as lactate and succinate, which are commonly found in diabetic wounds, drive additional PTMs, including lactylation and succinylation, respectively [45, 46]. These PTMs may interact synergistically or antagonistically with O-GlcNAc modification to impact protein stability, enzymatic activity, and signalling cascades, ultimately influencing complex metabolic crosstalk that contributes to DFU pathogenesis. Thus, a comprehensive analysis of the interactions among O-GlcNAcylation, phosphorylation, and other PTMs is crucial to gain deeper insights into the molecular mechanisms underlying DFUs.

### O-GlcNAc modification in the pathogenesis of DFUs

####  O-GlcNAc modification and diabetic peripheral neuropathy

**Figure 2:**
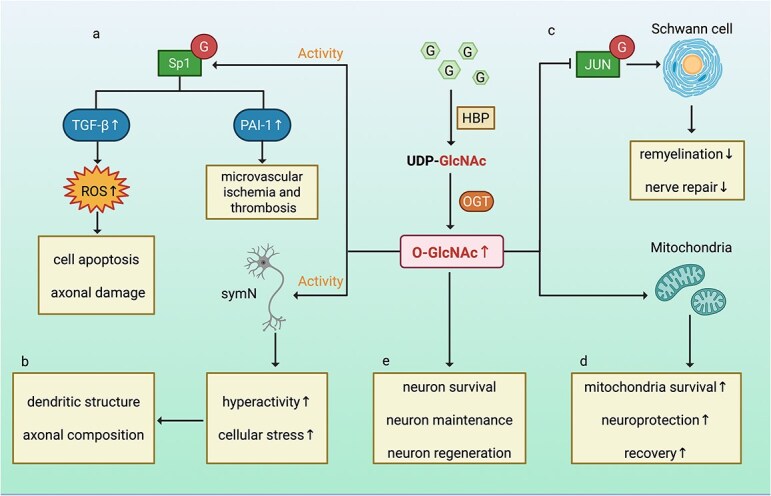
Role of O-GlcNAc modification in DPN and its contribution to the pathogenesis of DFUs. (**a**) High glucose enhances the O-GlcNAc modification and activation of Sp1, leading to increased glucose-induced gene expression of PAI-1 and TGF-β. this process contributes to microvascular ischemia and thrombosis in DPN and accelerates cell apoptosis and axonal damage by promoting the production of ROS. (**b**) Increased O-GlcNAc modification under high glucose conditions promotes symN hyperactivity and cellular stress, which can be alleviated by inhibiting O-GlcNAc modification with an OGT inhibitor. This leads to alterations in dendritic structure and axonal composition, which have been suggested as the cause of DPN-associated autonomic neuropathy. (**c**) In Schwann cells, O-GlcNAc modification promotes the OGlcNAc modification and activation of proteins, resulting in decreased JUN activity. This impairs the remyelination and nerve repair function of SCs. (**d**) O-GlcNAc-modified mitochondria enhanced mitochondria survival, neuroprotection and recovery. (**e**) O-GlcNAc signaling mediates sensory neuron survival, maintenance, and regeneration after injury. *DPN* diabetic peripheral neuropathy, *DFUs* Diabetic foot ulcers, *Sp1* specificity protein 1, *PAI-1* plasminogen activator inhibitor-1, *TGF-β* transforming growth factor-β, *ROS* reactive oxygen species, *symN* Sympathetic neuron, *HBP* hexosamine biosynthetic pathway, *OGT* O-GlcNAc transferase

DPN is a common chronic complication of diabetes characterized by distal symmetric polyneuropathy (DSPN) and diabetic autonomic neuropathy (DAN) [47]. Hyperglycaemia, dyslipidaemia, insulin resistance, and microvascular disorders contribute to DPN development through various pathways, such as the protein kinase C (PKC) pathway, the polyol pathway, the advanced glycation end products (AGE) pathway, and the hexosamine pathway. These pathways lead to inflammation, oxidative stress, and mitochondrial dysfunction, ultimately disrupting peripheral nerve function [48, 49]. Recent studies have highlighted the role of O-GlcNAc modification in DPN. Elevated O-GlcNAc levels have been observed in diabetic conditions, particularly in tissues affected by neuropathy. O-GlcNAc modification influences transcription factors and cellular signalling pathways that contribute to nerve damage. Under hyperglycaemic conditions, the flux of O-GlcNAc modification increases, which activates the specificity protein 1 (Sp1) pathway [50]. Increased O-GlcNAc modification of Sp1 under hyperglycaemic conditions enhances the expression of certain genes, such as plasminogen activator inhibitor-1 (PAI-1) and transforming growth factor-β (TGF-β), in several cell lines [51] (Figure 2a). PAI-1 overexpression is implicated in microvascular ischaemia and thrombosis in DPN patients [52]. TGF-β has been shown to induce cell apoptosis and axonal damage by promoting ROS production in human lens epithelial cells [53]. Functional defects in postganglionic sympathetic neurons (symNs), characterized by alterations in dendritic structure and axonal composition, have been suggested as the cause of DPN-associated autonomic neuropathy (DAN) [54]. Studies have revealed that increased O-GlcNAc modification under high-glucose conditions leads to symN overactivity in patients and animal models of diabetes mellitus. Furthermore, the inhibition of O-GlcNAc modification with the Ac4-5SGlcNAc (Ac5S) OGT inhibitor alleviates high glucose-induced symN hyperactivity and cellular stress [22] (Figure 2b). This finding suggests that diabetic DAN may be treated in the future by regulating O-GlcNAc modification to normalize symN activity.

O-GlcNAc modification has been shown to affect Schwann cell function. Schwann cells (SCs), the myelinating glial cells of the peripheral nervous system, play a vital role in nerve development, structural maintenance, and function. SCs are instrumental in promoting nerve repair and regeneration [55]. Metabolic dysfunctions in SCs contribute to the development of DPN. Impaired O-GlcNAc cycling in SCs leads to disrupted remyelination and impaired nerve repair in individuals with DPN. The JUN transcription factor, a key regulator in the SC injury response, is modified by O-GlcNAc at multiple sites. A decrease in O-GlcNAc modification leads to increased JUN activity, impairing the repair function of SCs in mice [56] (Figure 2c). In a mouse model of stroke, O-GlcNAc modification of extracellular mitochondria has been shown to reduce neuronal damage and improve neurological deficits. This process, known as mitochondrial transfer, where mitochondria enter neighbouring cells through intercellular junctions in the central nervous system (CNS), may protect neurons from injury and disease [57]. Modifying mitochondria with O-GlcNAc modification may prevent mitochondrial protein glycation and increase the viability of mitochondria when transferred into neurons, thereby improving the therapeutic efficacy of mitochondrial transfer in the CNS [58] (Figure 2d). Experiments in *Caenorhabditis elegans* have demonstrated that disrupting OGT or OGA through *in vivo* laser axotomies, which reduce or increase O-GlcNAc levels, respectively, significantly promotes neuronal regeneration. O-GlcNAc signalling mediates the cellular trophic status, coordinating the metabolism of damaged neurons and maximizing regenerative responses [59]. Furthermore, sensory neuron-specific knockout of OGT in mice results in reduced sensitivity to thermal and mechanical stimuli, accompanied by decreased epidermal innervation and loss of cell bodies in the dorsal root ganglia, and these neurons also show reduced axonal outgrowth [60]. Impaired O-GlcNAc cycling is also implicated in the pathogenesis of several neurodegenerative diseases, such as Alzheimer’s disease and Parkinson’s disease [61]. O-GlcNAc modification is essential for sensory neuron survival, maintenance, and regeneration after injury (Figure 2e). These findings significantly broaden the understanding of how metabolic changes, such as those occurring in diabetes and its complications, interact with neurological function.

####  O-GlcNAc modification and peripheral arterial disease

DPN and PAD interact synergistically in the pathogenesis of diabetic foot, significantly contributing to its onset and progression. This interaction encompasses various aspects, such as metabolic dysregulation, oxidative stress, inflammation, microvascular alterations, and neurovascular injury. Collectively, these factors drive the intricate pathological mechanisms underlying diabetic foot complications. PAD, characterized by insufficient blood perfusion due to stenosis or obstruction of blood vessels in the extremities, is implicated in up to 50% of DFUs [62]. Atherosclerosis is the main pathological process underlying PAD [63]. This condition leads to vessel wall thickening, stenosis, and calcification. Additionally, plaque rupture can result in peripheral arterial thrombosis, especially in individuals with diabetes, directly causing arterial occlusion and lower limb ischaemia, which subsequently contributes to DFU formation. When arterial perfusion to the foot is inadequate to maintain the functional integrity of the skin, ischaemic ulcers or gangrene may develop, particularly with occlusion of distal arteries, such as the dorsalis pedis artery [64]. Both diabetic microangiopathy and macroangiopathy arise from a combination of irreversible complex nonenzymatic glycation, elevated oxidative stress, inflammation, endothelial dysfunction, and a hypercoagulable state [65].

**Figure 3:**
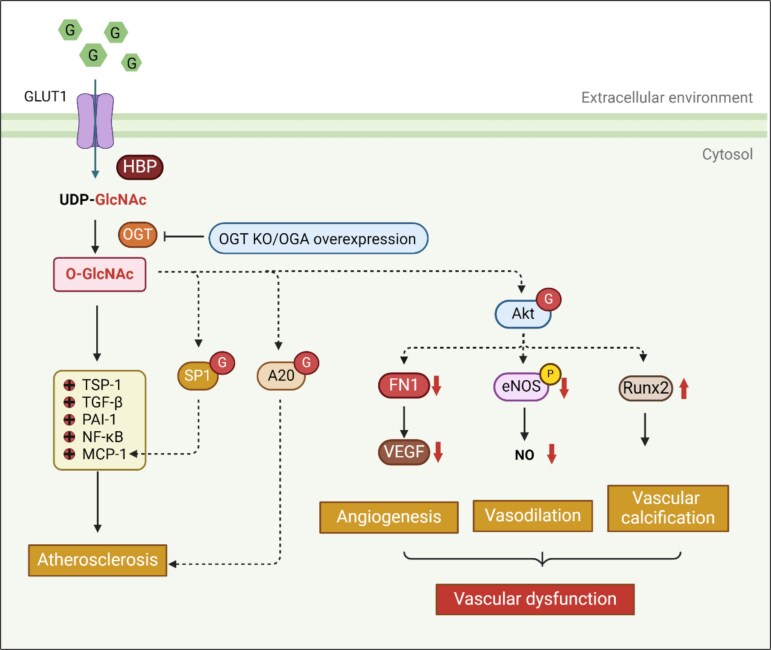
O-GlcNAc modification plays a mediating role in atherosclerosis and vascular dysfunction. O-GlcNAc modification promotes the upregulation of TSP-1, TGF-β, PAI-1, and NF-κB genes associated with atherosclerosis in vascular smooth muscle cells and endothelial cells. An increase in O-GlcNAc modification of the transcription factor Sp1 reduces its degradation, thereby enhancing the transcription of the pro-atherogenic gene MCP-1. O-GlcNAc acetoxylation promotes the ubiquitin-proteasome degradation of A20, namely tumor necrosis factor alpha-induced protein 3 (TNFAIP3), accelerating atherosclerosis in diabetic mice. O-GlcNAc modification-mediated AKT O-GlcNAc modification and activation leads to downregulating eNOS and VEGF, reducing vasodilation and angiogenesis. It also increases the upregulation of Runx2, thereby leading to vascular calcification and promoting the occurrence and development of vascular dysfunction. OGT deficiency reduces atherosclerotic lesions in hyperglycemic smOGT knockout mice. OGA overexpression reversed coronary endothelial dysfunction. *GLUT1* glucose transporter type 1, *HBP* hexosamine biosynthetic pathway, *OGT* O-GlcNAc transferase, *OGA* O-GlcNAcase, *TSP-1* thrombospondin-1, *TGF-β* transforming growth factorbeta, *PAI-1* plasminogen activator inhibitor-1, *NF-κB* nuclear factor kappa-light-chain-enhancer of activated B cells, *Sp1* specificity protein 1, *MCP-1* monocyte chemoattractant protein-1, *A20* atheroprotective protein A20, *AKT* protein kinase B, *FN1* fibronectin 1, *eNOS* endothelial nitric oxide synthase, *VEGF* vascular endothelial growth factor, *Runx2* runt-related transcription factor 2

O-GlcNAc modification plays a critical role in the development of atherosclerosis through multiple mechanisms. Extensive research has shown that O-GlcNAc modification is a significant pathogenic factor in diabetic vascular complications. Previous studies have demonstrated that the cultivation of vascular cells, including vascular smooth muscle cells (VSMCs) and endothelial cells, in high-glucose medium leads to a continuous increase in O-GlcNAc modification (Figure 3). This modification mediates the upregulation of numerous genes associated with atherosclerosis, such as thrombospondin-1 (TSP-1), TGF-β, plasminogen activator inhibitor-1 (PAI-1), and nuclear factor kappa-light-chain-enhancer of activated B cells (NF-κB) [66]. Additionally, increased O-GlcNAc modification of the Sp1 transcription factor stabilizes Sp1, enhancing the transcription of the monocyte chemoattractant protein-1 (MCP-1) pro-atherogenic gene [67]. The A20 anti-inflammatory and atheroprotective protein is also a target of O-GlcNAc modification; O-GlcNAc modification of A20 promotes ubiquitin–proteasome degradation, thereby accelerating atherosclerosis in diabetic ApoE-null mice [68]. O-GlcNAc modification activates AKT, leading to the upregulation of Runt-related transcription factor 2 (Runx2) and promoting the osteogenic differentiation of VSMCs, which contributes to vascular calcification [69]. Furthermore, smooth muscle OGT deficiency reduces atherosclerotic lesions in hyperglycaemic mice fed a Western diet, highlighting the role of OGT-mediated O-GlcNAc modification in atherosclerosis under hyperglycaemic conditions [24].

O-GlcNAc modification impairs vascular function through several mechanisms, including effects on angiogenesis, vasodilation, and vascular calcification. O-GlcNAc modification inhibits the expression and function of vascular endothelial growth factor (VEGF) via the suppression of AKT, thereby suppressing angiogenesis. O-GlcNAc modification also inhibits endothelial nitric oxide synthase (eNOS) activity, leading to reduced vasodilation [25]. In coronary endothelial cells isolated from type 1 diabetic mice, low OGA expression and high OGT expression are associated with impaired endothelium-dependent vasodilation; however, OGA overexpression reverses this dysfunction [70]. O-GlcNAc modification promotes vascular calcification by activating AKT and upregulating Runx2, driving the osteogenic differentiation of VSMCs [69]. In conclusion, increased O-GlcNAc modification promotes atherosclerosis and damage vascular function through mechanisms involving gene regulation, inflammation, and direct effects on VSMCs and endothelial cells. These alterations may contribute to the formation and progression of DFUs.

### O-GlcNAc modification in the healing process of DFUs

####  O-GlcNAc modification and persistent hyperinflammation in DFUs

The series of complications arising from DPN and PAD, both of which are complications of diabetes, can predispose patients to foot ulcers. When local bacterial and/or fungal invasions occur, diabetic foot infection (DFI) can develop, potentially progressing to severe osteomyelitis. Globally, the predominant pathogens responsible for DFI are gram-positive bacteria, with *Staphylococcus aureus* being the most common, followed by *Pseudomonas aeruginosa* and *Escherichia coli* [71]. Pathogens can form biofilms on diabetic foot wounds, creating a protected environment resistant to antibiotics, thus playing a critical role in the development and persistence of DFU infections [72].

**Figure 4:**
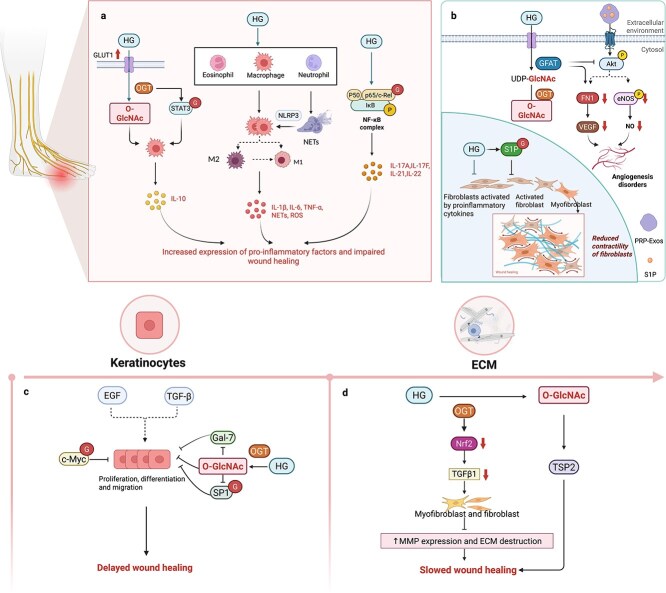
O-GlcNAc modification and poor wound healing in DFU. (**a**) Increasing glucose supply or GLUT1 expression can enhance protein O-GlcNAc modification, which can negatively regulate IL-10 gene expression in macrophages via STAT3 modification. High glucose levels promote the infiltration of macrophages, neutrophils, and other immune cells, driving macrophages toward the pro-inflammatory M1 phenotype while reducing the transition to the anti-inflammatory M2 phenotype. This shift increases proinflammatory factor expression and decreases anti-inflammatory factor expression. Additionally, long-term over-activation and dysregulated apoptosis of neutrophils can lead to the formation and release of NETs, which activate the NLRP3 inflammasome in macrophages, resulting in IL-1β release. High glucose levels also augment O-GlcNAc modification, promoting NF-κB activation, which in turn induces pathological overactivation of lymphocytes and macrophages, leading to increased production of pro-inflammatory mediators and an exacerbated inflammatory response. (**b**) Hyperglycemia leads to increased O-GlcNAc modification in endothelial cells, which in turn decreases Akt-mediated phosphorylation of eNOS. This reduction in eNOS phosphorylation results in lower NO production and contributes to endothelial dysfunction. However, inhibiting GFAT can counteract the negative effects of hyperglycemia on eNOS phosphorylation and enhance VEGF-induced angiogenesis. Moreover, O-GlcNAc acts as a molecular buffer to modulate S1P signaling, ultimately causing fibroblast contraction. (**c**) EGF and TGF-β promote the proliferation and migration of keratinocytes at the wound edge, which is crucial for re-epithelialization. However, in diabetic wounds, O-GlcNAc modification of c-Myc significantly contributes to keratinocyte dysfunction. Inhibiting c-Myc and its O-GlcNAc modification can help alleviate delays in keratinocyte migration, proliferation, and differentiation. High glucose levels can exacerbate this issue by increasing O-GlcNAc modification, which inhibits Sp1 transcription and reduces Gal-7 expression, ultimately hindering keratinocyte differentiation and migration. (**d**) O-GlcNAc glycosylation has been identified as a factor that enhances the expression of TSP2, which contributes to impaired wound healing in diabetes. Additionally, O-GlcNAc modification inhibits Nrf2 signaling, leading to a significant decrease in TGF-β1 levels. This reduction in TGF-β1 results in the up-regulation of MMPs, which degrade the ECM and thereby hinder the healing process of diabetic wounds. *GLUT1* glucose transporter type 1, *STAT3* signal transducer and activator of transcription 3, *IL* interleukin 10, *TNF-α* tumor necrosis factor-alpha, *NETs* neutrophil extracellular traps, *ROS* reactive oxygen species, *NF-κB* nuclear factor kappa-light-chain-enhancer of activated B cells, *Akt* protein kinase B, *FN1* fibronectin 1, *eNOS* endothelial nitric oxide synthase, *NO* nitric oxide, *GFAT Glutamine* Fructose-6-phosphate amidotransferase, *OGT* O-GlcNAc transferase, *VEGF* enhance vascular endothelial growth factor, *S1P* sphingosine-1-phosphate, *EGF* epidermal growth factor, *TGF- β* transforming growth factor-beta, *c-Myc* v-myc avian myelocytomatosis viral oncogene homolog, *SP1* specificity protein 1, *Gal-7* galectin-7, *TSP2* thrombospondin-2, *Nrf2* nuclear factor erythroid 2-related factor 2, *ECM* extracellular matrix, *MMP* matrix metalloproteinase

Inflammation in patients with DFUs is characterized by a persistent and excessive inflammatory response. OGT-mediated O-GlcNAc modification exacerbates inflammation through various mechanisms under hyperglycaemic conditions, as high blood sugar disrupts the immune system and impairs the immune response [73]. Glucose transporter 1 (GLUT1) is a primary transporter in certain immune cells, and increasing the glucose supply or GLUT1 expression enhances UDP-GlcNAc production, thereby increasing protein O-GlcNAc modification on the hepatocyte surface [74] (Figure 4a). Signal transducer and activator of transcription 3 (STAT3) is a pivotal transcription factor that promotes inflammation and tissue repair [75]. Studies have indicated that O-GlcNAc modification of the Thr717 transcriptional activation domain of STAT3 negatively regulates the phosphorylation of STAT3 and affects interleukin 10 (IL-10) gene expression in macrophages, thus maintaining a hyperinflammatory state [76]. Diabetic patients often exhibit abnormal glucose metabolism, which can promote the production and release of inflammatory mediators, thereby leading to the infiltration of macrophages, neutrophils, and other immune cells. Long-term exposure to high glucose levels or increased expression of GLUT1 promotes the transformation of macrophages into the M1 phenotype [77]. In individuals with DFUs, the presence of increased M1 macrophages and proinflammatory factor expression, coupled with decreased M2 macrophages and anti-inflammatory factor expression, results in a predominance of M1 macrophages that maintain a proinflammatory state. The failure of macrophages to transition from the M1 to the M2 phenotype may be a key issue in the impaired wound healing observed in diabetes [78, 79]. This results in an environment rich in inflammatory factors, such as interleukin 1beta (IL-1β), interleukin 6 (IL-6), and tumour necrosis factor-alpha (TNF-α) [80]. M1 macrophages also promote oxidative stress reactions, generating reactive oxygen species (ROS) that cause tissue damage [81]. Studies have reported a dual dysfunction of neutrophils in diabetic patients; recruitment, chemotaxis, and phagocytosis are decreased, and the ability of neutrophils to control macrophage polarization is diminished. These dysfunctions impair the immune response to infection [82, 83]. In diabetic wounds, long-term overactivation of neutrophils and dysregulated apoptosis in neutrophils promote the formation and release of neutrophil extracellular traps (NETs), which activate the NLRP3 inflammasome in macrophages and lead to the subsequent release of IL-1β. This results in an enhanced inflammatory response and delayed wound healing [84]. Studies have reported that elevated levels of intracellular O-GlcNAc modification increases IL-1β expression in M1 macrophages but have a minimal effect on arginase 1 expression in M2 macrophages [85]. These findings suggest that O-GlcNAc modification amplifies the proinflammatory activity of M1 macrophages. NF-κB, which has been extensively studied, is modified by O-GlcNAc, and it is one of the most critical transcription factors in macrophages and a key player in inflammatory signalling pathways [86, 87]. Growing evidence supports the critical role of O-GlcNAc modification in NF-κB activation in B and T cells, with c-Rel being the predominant O-GlcNAcylated NF-κB subunit in lymphocytes. Under high glucose conditions, increased O-GlcNAc modification augments the transcriptional activity of c-Rel, leading to pathological overactivation of lymphocytes and autoimmune responses through the production of helper T cell cytokines [88], particularly in the Th17 cell subset, which secretes proinflammatory cytokines, including interleukin-17A, interleukin-17F, interleukin-21, and interleukin-22 [89]. High glucose levels augment O-GlcNAc modifications, promoting NF-κB activation, which in turn induces macrophages to produce TNF-α and other proinflammatory mediators, thereby exacerbating the inflammatory response [90]. All of these mechanisms contribute to the long-term maintenance of high levels of inflammatory factors, keeping the wound in a state of chronic inflammation. Consequently, the function of fibroblasts and vascular endothelial cells is downregulated, and the formation of granulation tissue is inhibited.

####  O-GlcNAc modification and impaired granulation tissue formation in DFUs

Granulation tissue is composed of new capillaries formed by endothelial cells and proliferating fibroblasts [91, 92]. Hyperglycaemia-induced endothelial dysfunction is a key link and initiating factor in the pathophysiological basis and development of diabetic vasculopathy [93]. In patients with DFUs, insufficient or dysfunctional neovascularization primarily affects the proliferative phase of wound healing [94]. The long-term high-glucose microenvironment stimulates various conditions, including glucose metabolism disorders, lipid metabolism disorders, insulin resistance, inflammatory responses, oxidative stress, mitochondrial damage, and the activation of AGEs and their receptors. These conditions contribute to vascular endothelial damage, reduce the expression of VEGF and other growth factors, and limit angiogenesis, thus impeding progression to the proliferative phase of wound healing [95].

Hyperglycaemia drives elevated O-GlcNAc modification levels in endothelial cells. Increased O-GlcNAc modification It leads to a decrease in Akt-mediated phosphorylation of eNOS, which in turn reduces nitric oxide (NO) production and promotes endothelial dysfunction in isolated endothelial cells obtained from the forearm vein of patients with T2DM [96] (Figure 4b). Inhibition of the GFAT rate-limiting enzyme of the HBP reverses the inhibitory effect of hyperglycaemia on eNOS phosphorylation in cultured endothelial cells [97]. These findings suggest that inhibiting O-GlcNAc modification could be restored in diabetic patients. Previous studies have shown that pharmacological inhibition or siRNA-mediated downregulation of GFAT1 enhances VEGF-induced angiogenesis, indicating that GFAT1 acts as a negative regulator of angiogenesis. Specifically, activating GFAT1/HBP under high-glucose conditions impairs vessel sprouting, whereas inhibiting GFAT1 improves angiogenesis even under high-glucose conditions. This insight provides a novel understanding of how the HBP influences angiogenesis [98]. Additionally, increased O-GlcNAc modification has been shown to enhance the vascular response to constrictive stimuli [99]. In summary, O-GlcNAc modification can cause endothelial cell dysfunction, vasoconstriction, and impaired neovascularization under hyperglycaemic conditions, which can lead to reduced blood flow, local ischaemia, and hypoxia, potentially causing or exacerbating foot ulcers and necrosis.

Fibroblasts proliferate, migrate into the matrix formed by fibrin clots, secrete extracellular matrix (ECM) components, and differentiate into myofibroblasts to form granulation tissue. Myofibroblasts express actin to promote wound contraction and remodelling [100, 101]. Hyperglycaemic conditions can lead to decreased fibroblast proliferation, increased apoptosis, impaired function, and inhibited migration to wounds, resulting in impaired repair and delayed healing of diabetic wounds [102]. Sphingosine-1-phosphate (S1P) is a crucial lipid signalling molecule that induces collagen matrix contraction in fibroblasts [103]. O-GlcNAc acts as a molecular buffer that controls S1P signalling, ultimately leading to fibroblast contraction [104]. Fibroblasts with low O-GlcNAc levels exhibit increased sensitivity to S1P. Studies have shown that compared with untreated fibroblasts, mouse fibroblasts treated with Thiamet-G, an OGA inhibitor, present elevated O-GlcNAc levels and are more resistant to S1P signal-related contractions [105]. In contrast to cells cultured in low glucose, cells cultured in high glucose lack sensitivity to S1P-mediated contraction [104]. These findings suggest that high levels of O-GlcNAc are unfavourable for fibroblast contraction and wound healing. Additionally, S1P is a key regulator of vascular homeostasis and angiogenesis, and it is highly enriched in exosomes derived from platelet-rich plasma (PRP-Exos) [106, 107]. Recently, researchers have reported that under high-glucose conditions, PRP-Exos transfer S1P signals and activate the AKT/FN1 pathway by binding to S1P receptor 1 on the membrane of vascular endothelial cells, which promotes cell proliferation, migration, angiogenesis, and wound closure in diabetic wounds. These findings provide a preliminary theoretical basis for the potential use of PRP-Exos in the treatment of DFUs [107].

####  O-GlcNAc modification and re-epithelialization impairment in DFUs

During the healing stage of DFUs, wound epithelialization and dermal repair are vital for skin tissue regeneration. Research has indicated that after granulation tissue forms, several growth factors, such as epidermal growth factor (EGF) and TGF-β, stimulate the proliferation and migration of keratinocytes at the wound edge, facilitating re-epithelialization (Figure 4c). Subsequently, keratinocytes proliferate and differentiate to restore the integrity and function of the epidermis. Dermal repair primarily involves the proliferation, differentiation, and secretion of fibroblasts. Throughout the healing process, keratinocytes interact with various cell types to synergistically promote efficient wound healing [108]. Under hyperglycaemic conditions, keratinocytes exhibit reduced proliferation, differentiation, and migration capabilities. These impairments in keratinocyte functions are likely to lead to inadequate re-epithelialization, which also provides a plausible explanation for the difficulty in healing DFUs [109].

Research has indicated that hyperglycaemia delays keratinocyte advancement in wound healing by increasing intracellular protein O-GlcNAc modification. OGT knockdown accelerates wound healing under both normal and hyperglycaemic conditions. Targeted inhibition of OGT alters the rate of wound closure, suggesting that inhibiting OGT promotes wound healing in diabetic skin [110]. C-Myc, a transcription factor that regulates genes related to migration, proliferation, differentiation, and apoptosis, is increased in keratinocytes at the edges of chronic wounds. C-Myc positivity in wound-edge keratinocytes serves as a marker of nonhealing wounds. O-GlcNAc modification of c-Myc plays a significant role in keratinocyte dysfunction in diabetic wounds. Inhibition of c-Myc and its O-GlcNAc modification alleviates the delay in diabetic wound healing [26]. O-GlcNAc modification has been identified as a crucial regulatory mechanism for keratinocyte differentiation [111]. Sp1, an O-GlcNAcylated protein and important transcription factor, is also involved in keratinocyte differentiation. OGT overexpression specifically inhibits the transcriptional activation of Sp1 in keratinocytes [112, 113]. Thus, it is plausible that the increase in O-GlcNAc modification caused by high glucose may ultimately hinder keratinocyte differentiation by inhibiting Sp1 transcription. Additionally, loss of galectin-7 (Gal-7) impairs keratinocyte migration and re-epithelialization of skin wounds. Enhanced O-GlcNAc glycosylation in a high-glucose environment significantly reduces Gal-7 levels in keratinocytes, thereby affecting their function [114].

#### O-GlcNAc modification and ECM degradation in DFUs

The ECM plays a pivotal role in coordinating the healing process by providing structural support and facilitating cell–cell and cell–matrix interactions. In diabetes, ECM damage can disrupt these interactions, resulting in delayed healing [115]. Thrombospondin 2 (TSP2), a matricellular protein that influences cell–matrix interactions, is an ECM component integral to wound healing. TSP2 knockout mice display accelerated wound healing, characterized by altered ECM composition and increased vascular density [116]. Recent experimental findings have suggested that hyperglycaemia-induced TSP2 expression contributes to impaired wound healing in diabetes. O-GlcNAc modification has been reported to increase TSP2 expression levels, which contributes to impaired healing in diabetes [117] (Figure 4d).

Fibroblasts that express high levels of matrix metalloproteinases (MMPs) cause excessive ECM degradation and destruction of collagen, fibronectin and other proteins, and they affect the normal formation of the collagen matrix and hinder the restoration of diabetic foot wounds [118]. Compared with those in normal wounds in patients without diabetes, the levels of MMP-1, MMP-8, MMP-9, and activated MMP-2 are significantly increased in DFUs [119]. Inhibition of MMP-2 promotes wound healing in diabetic mice [120]. A recent study has revealed that the activation of nuclear factor erythroid-2-related factor-2 (Nrf2) signalling significantly upregulates TGF-β1 and downregulates MMP9 during diabetic wound healing [121]. Nrf2 is a key transcription factor in the cellular defence system that regulates the expression of antioxidant genes in response to pro-oxidative and proinflammatory stresses [122]. Recent studies have shown that the inhibition or gene knockdown of OGT induces the activation of the Nrf2 pathway [123]. O-GlcNAc modification may lead to increased MMP9 levels through this pathway. Additionally, research has shown that increased O-GlcNAc modification upregulates MMP-1, MMP-2, and MMP-3 expression, thereby promoting the migration of tumour cells [124]. O-GlcNAc modification may degrade the ECM after MMPs are upregulated, thereby hindering diabetic wound healing.

### O-GlcNAc modification and metabolic regulatory factors in and out of DFUs

#### O-GlcNAc modification and oxidative stress in DFUs

**Figure 5:**
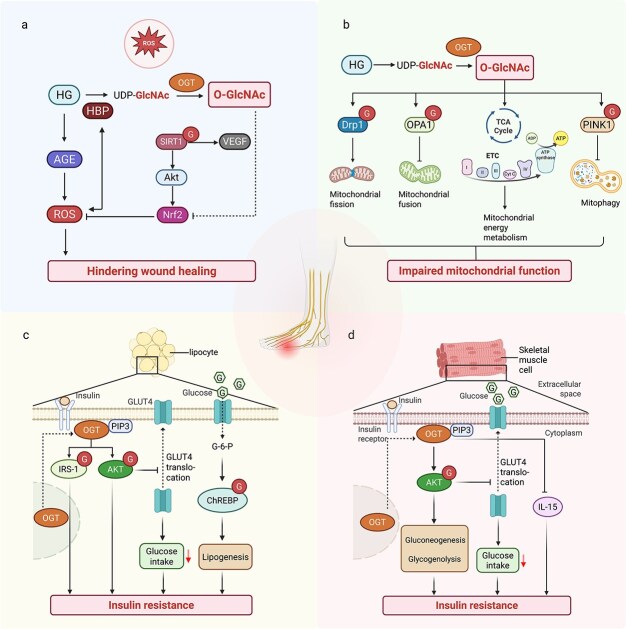
High glucose modulates O-GlcNAc modification of metabolic regulatory factors both in and out DFUs. (**a**) Hyperglycemia induces the formation of AGEs in the blood, which directly leads to excessive ROS production. The activation of the HBP can also induce ROS production and oxidative stress; conversely, ROS can activate the HBP. Additionally, hyperglycemia inhibits Nrf2 activation, resulting in oxidative stress in macrophages. Activation of SIRT1 protects vascular endothelial cells from oxidative stress damage via the AKT-Nrf2 pathway, thereby improving wound angiogenesis and accelerating wound healing. (**b**) O-GlcNAc modification affects mitochondrial dynamics, including fusion and fission processes. Drp1 is O-GlcNAcylated at specific sites, promoting its translocation to mitochondria and driving mitochondrial division. Additionally, high-glucose conditions can increase O-GlcNAc modification of OPA1, leading to reduced mitochondrial fusion. O-GlcNAc modification regulates mitochondrial energy metabolism by modulating the TCA cycle，the ETC, and oxidative phosphorylation. Long-term high glucose leads to O-GlcNAc over-modification, which leads to decreased ATP synthesis, increased oxidative stress, and insufficient cellular energy by affecting the TCA cycle, ETC, and oxidative phosphorylation. O-GlcNAc modification can regulate mitophagy through a PINK1-dependent pathway. Increased O-GlcNAc modification can impair mitophagy, leading to the accumulation of damaged mitochondria and exacerbating cellular stress. In addition, O-GlcNAc modification can indirectly promote DFUs progression by inducing IR. Increased O-GlcNAc modification level is associated with IR in multiple tissues, such as adipose tissue and skeletal muscle. (**c**) When the duration of insulin stimulation is too long, OGT will transfer from the nucleus of adipocytes to the cytoplasm and bind to PIP3 to localize to the cell membrane. Activation of multiple key factors, thereby altering the insulin signaling pathway. The activation of AKT leads to the block of GLUT4 translocation and the decrease of glucose uptake in adipocytes, which in turn leads to insulin resistance. OGT reduces IRS-1 activity by inhibiting its phosphorylation, leading to IR. In addition, the increase of O-GlcNAc modification under a high glucose environment can increase the protein content and transcriptional activity of ChREBP, promote lipid synthesis, and eventually lead to IR. (**d**) In skeletal muscle, the up-regulation of O-GlcNAc modification activates AKT through the insulin signal transduction pathway, leading to IR by promoting gluconeogenesis, glycogenolysis, and inhibiting glucose uptake. OGT knockdown increases IL-15 production in skeletal muscle, thereby improving insulin sensitivity. *AGE* advanced glycation end product, *ROS* reactive oxygen species, *HBP* hexosamine biosynthetic pathway, *SIRT1* sirtuin 1, *AKT* protein kinase B, *Nrf2* nuclear factor erythroid 2-related factor 2, *VEGF* enhance vascular endothelial growth factor, *Drp1* dynamin-related protein 1, *OPA1* optic atrophy 1, *TCA* tricarboxylic acid cycle, *ETC* electron transport chain, *ATP* adenosine triphosphate, *PINK1* PTEN-induced kinase 1, *PIP3* phosphatidylinositol-3,4,5-trisphosphate, *GLUT4* Glucose transporter 4, *IRS-1* lnsulin receptor substrate-1, *ChREBP*, Carbohydrate response element binding protein, *OGT* O-GlcNAc transferase, *IL-15* interleukin-15

Excessive oxidative stress and decreased antioxidant capacity, which lead to redox imbalance, are also key contributors to the failure of diabetic wound healing. ROS are pivotal regulators of several stages of wound healing, with low levels being essential for combating external damage [125]. However, high oxidative stress, primarily due to excess ROS, often results in persistent inflammation [126]. After injury, ROS are produced by inflammatory cells, epithelial cells, and endothelial cells. In the context of DFUs, persistent hyperglycaemia leads to the formation of AGEs in the blood, which directly induces excessive ROS production [127] (Figure 5a). Furthermore, the activation of the polyol pathway due to elevated glucose levels results in ROS accumulation and oxidative stress in DFUs, which occurs by reducing the cellular antioxidant capacity and impairing the nutrient and oxygen supply to the cells involved in the wound-healing process [128]. Excessive ROS not only cause chronic inflammation but also impair angiogenesis, promote cellular senescence, hinder re-epithelialization, and induce microthrombosis, all of which negatively impact wound healing [129].

The activation of the HBP has been shown to induce ROS production and oxidative stress; conversely, ROS can also lead to HBP activation [130]. Most instances of HBP-induced oxidative stress are associated with hyperglycaemia [131]. Oxidative stress related to hyperglycaemia not only increases flux through the HBP and elevates O-GlcNAc levels but also amplifies the inflammatory response and impairs endothelial function [132]. *In vitro* studies have demonstrated that hyperglycaemia inhibits Nrf2 activation, leading to oxidative stress in rat macrophages [133]. Nrf2 has been confirmed to be an essential antioxidant in diabetic wound treatment [134]. OGT inhibition or gene knockdown activates the Nrf2 pathway, whereas sustained elevation of O-GlcNAc reduces the Nrf2-mediated oxidative stress response, potentially hindering wound healing [135]. Sirtuins, a family of NAD + -dependent deacetylases, act as cellular sensors to detect energy availability and regulate metabolic processes. Sirtuin 1 (SIRT1) and Sirtuin 3 (SIRT3), located in the nucleus and mitochondria, respectively, are critical for controlling metabolic processes [136]. Studies have reported that SIRT1 activation protects vascular endothelial cells from oxidative stress damage, improves wound angiogenesis, and accelerates wound healing in diabetic mice, possibly through the AKT–Nrf2 pathway [137]. Elevated O-GlcNAc modification of SIRT1 increases its deacetylase activity, thereby protecting cells from stress-induced apoptosis [138]. Additionally, recent studies have shown that SIRT3 deficiency reduces blood supply, inhibits VEGF expression, promotes superoxide production, and impairs total antioxidant capacity, leading to delayed skin wound healing in diabetic mice [139]. The interplay between O-GlcNAc modification and oxidative stress significantly impacts diabetic wound healing, contributing to impaired angiogenesis, inflammation, and overall wound repair under diabetic conditions.

#### O-GlcNAc modification and mitochondrial dysfunction in DFUs

O-GlcNAc modification plays a crucial role in regulating mitochondrial function and energy metabolism (Figure 5b). In the context of DFUs, mitochondrial dysfunction is a key pathological feature, and O-GlcNAc modification has emerged as a significant regulator of this dysfunction. O-GlcNAc modification affects mitochondrial dynamics, including fusion and fission processes. High levels of O-GlcNAc modification can lead to increased mitochondrial fragmentation, decreased mitochondrial membrane potential, and impaired mitochondrial function. For example, Drp1, a key protein involved in mitochondrial fission, is O-GlcNAcylated at specific sites, promoting its translocation to mitochondria and driving mitochondrial division in neuronal cells [140]. Additionally, high-glucose conditions increase O-GlcNAc modification of OPA1, a protein involved in mitochondrial fusion, leading to mitochondrial dysfunction in neonatal cardiac myocytes [141].

O-GlcNAc modification regulates mitochondrial energy metabolism by modulating the tricarboxylic acid (TCA) cycle and the electron transport chain (ETC). This modification affects the activity of mitochondrial respiratory chain complexes, leading to reduced ATP production and increased oxidative stress [142]. Studies have shown that O-GlcNAc modification of mitochondrial proteins involved in oxidative phosphorylation impairs mitochondrial function and contributes to cellular energy deficits in cardiovascular diseases [143].

Mitophagy, the selective degradation of damaged mitochondria, is a critical mechanism for maintaining mitochondrial quality. O-GlcNAc modification has been shown to regulate mitophagy through certain pathways, such as the PINK1-dependent pathway. Inhibition of O-GlcNAc modification increases mitophagy, thereby reducing mitochondrial dysfunction and cellular damage. Conversely, increased O-GlcNAc modification impairs mitophagy, leading to the accumulation of damaged mitochondria and exacerbating cellular stress in a mouse model of Alzheimer’s disease and neuroblastoma cell lines [144].

####  O-GlcNAc modification regulates insulin sensitivity and secretion outside DFUs

In addition to DFUs, O-GlcNAc modification can indirectly contribute to the progression of DFUs by regulating insulin sensitivity, synthesis and secretion [145]. Increased O-GlcNAc modification levels are associated with insulin resistance in multiple tissues, such as adipose tissue and skeletal muscle [146, 147]. A previous study has reported that OGT ablation in white adipocytes of mice fed a high-fat diet prevents diet-induced obesity and improves glycaemic control. OGT deletion also reduces circulating insulin levels and improves insulin resistance, glucose tolerance and insulin sensitivity; the liver is also improved by OGT deletion, as indicated by reduced lipid content [148]. In vivo, the O-GlcNAc modification-induced insulin resistance in adipocytes is caused mainly by the attenuation of insulin signalling (Figure 5c). When the duration of insulin stimulation is too long, OGT is transferred from the nucleus to the cytoplasm where it binds to phosphatidylinositol (3,4,5)-trisphosphate (PIP3), which is localized to the cell membrane. This activation of multiple key factors thereby alters the insulin signalling pathway [149]. The activation of AKT leads to the blockade of GLUT4 translocation and a decrease in glucose uptake in adipocytes, which in turn leads to insulin resistance. O-GlcNAc modification also impairs insulin signalling by altering insulin receptor substrate-1 (IRS-1) and inhibiting its phosphorylation [145]. In addition, studies have reported that upregulated O-GlcNAc modification of carbohydrate response element-binding protein (ChREBP) increases its protein content and transcriptional activity in a high-glucose environment, thereby promoting liver lipogenesis, causing abnormal lipid accumulation, and leading to insulin resistance. Hepatic ChREBP knockdown attenuates peripheral IR in rats. Skeletal muscle is considered one of the most critical insulin-sensitive tissues, and more than 80% of the insulin secreted by the human body is used to help skeletal muscle uptake and utilize glucose [150]. In skeletal muscle, global upregulation of O-GlcNAc modification activates AKT, which leads to IR by promoting gluconeogenesis and glycogenolysis, as well as inhibiting glucose uptake [151]. OGT deficiency results in increased whole-body energy expenditure, altered muscle matrix utilization, and increased skeletal muscle interleukin-15 (IL-15) production (Figure 5d). Elevated plasma IL-15 levels may change the ability to store and metabolize energy substrates, improving whole-body insulin sensitivity and increasing resistance to obesity [152]. Overall, hyperglycaemia induces O-GlcNAc modification of certain key transcription factors and cofactors, causing insulin resistance and promoting glycogenesis and lipogenesis. This, in turn, further increases glucose levels, forming a vicious cycle that exacerbates glucose toxicity effects and thus worsens the progression of diabetes and diabetic complications [153].

### Latest advances and future directions in O-GlcNAc modification

While the role of O-GlcNAc modification in diabetic complications is increasingly recognized, recent advances highlight its potential as a therapeutic target. O-GlcNAc glycosylation is implicated in various pathological processes, including inflammation, oxidative stress, and metabolic dysregulation. Targeting O-GlcNAc modification is a novel therapeutic strategy for DFUs and other diabetic complications. For example, inhibiting OGT or enhancing OGA activity has shown promise in preclinical models, suggesting that modulating the O-GlcNAc cycle could mitigate diabetic complications [154]. However, translating these findings into clinical practice faces several challenges, including the need for selective inhibitors and a better understanding of tissue-specific O-GlcNAc functions.

Despite significant progress, several technical challenges remain in the study of O-GlcNAc modification. The dynamic and reversible nature of O-GlcNAc modification makes it difficult to capture and quantify O-GlcNAc modification *in vivo*. Recent advances in mass spectrometry and glycoproteomics have improved the detection and characterization of O-GlcNAc sites, but challenges persist in identifying low-abundance and tissue-specific modifications [155]. Additionally, the development of tools for site-specific O-GlcNAc modification in cells remains an active area of research. Breakthroughs in these areas could increase the ability to study O-GlcNAc modification in disease models and develop targeted therapies.

Future research on O-GlcNAc modification should focus on several key areas. First, there is a need for more comprehensive studies on the crosstalk between O-GlcNAc modification and other PTMs, such as phosphorylation and ubiquitination. This crosstalk is critical for regulating cellular signalling and could reveal new therapeutic targets. Second, the development of advanced imaging and detection techniques, such as nanopore sequencing and fluorescent labelling, will enable real-time monitoring of O-GlcNAc dynamics in living cells. Third, exploring the role of O-GlcNAc modification in emerging areas, such as mitochondrial function and autophagy, could provide new insights into its involvement in metabolic diseases. Finally, addressing the limitations of current studies, such as the lack of human clinical trials, will be essential for translating basic research findings into effective treatments. In conclusion, O-GlcNAc modification holds great promise for understanding and treating diabetic complications. Continued research into its mechanisms, clinical applications, and technological advancements will be crucial for realizing its full potential as a therapeutic target.

## Conclusions

Abnormal activation of O-GlcNAc modification is implicated in the pathogenesis of DFUs and hinders the healing of these wounds. Elevated levels of O-GlcNAc can induce microvascular ischaemia and impair nerve cell function. Furthermore, increased O-GlcNAc modification may promote atherosclerosis and vascular dysfunction, potentially leading to the formation and progression of DFUs. This modification can also maintain a hyperinflammatory state in DFU wounds. Under high-glucose conditions, O-GlcNAc modification impedes DFU wound healing by affecting neovascularization and endothelial cell dysfunction, impeding fibroblast contraction, disrupting keratinocyte function, blocking ECM remodelling, and inducing oxidative stress. Scientists are actively exploring methods to modulate O-GlcNAc modification to treat various diseases; OGT may be a promising drug target for promoting DFU wound healing. Future research should focus on small-molecule inhibitors that precisely modulate OGT and OGA, potentially affecting cellular functions through the addition or removal of O-GlcNAc modifications on target proteins. Ultimately, therapies that regulate O-GlcNAc modification may become more precise and effective. Additionally, we can further explore the relationship between O-GlcNAc modification and other cells involved in DFU wound healing, such as stem cells, and clarify how O-GlcNAc modification specifically regulates signalling pathways related to DFUs. Elucidating the mechanisms of O-GlcNAc modification in DFUs will provide new perspectives on the role of O-GlcNAc modification and offer new therapeutic ideas and methods for promoting the healing of diabetic foot wounds. Moreover, the relationship between O-GlcNAc modification and DFUs can be further studied to provide more insights for the comprehensive understanding and treatment of diabetes and its complications.
